# Thymoquinone induces apoptosis and DNA damage in 5-Fluorouracil-resistant colorectal cancer stem/progenitor cells

**DOI:** 10.18632/oncotarget.27426

**Published:** 2020-08-04

**Authors:** Farah Ballout, Alissar Monzer, Maamoun Fatfat, Hala El Ouweini, Miran A. Jaffa, Rana Abdel-Samad, Nadine Darwiche, Wassim Abou-Kheir, Hala Gali-Muhtasib

**Affiliations:** ^1^Department of Biology, American University of Beirut, Lebanon; ^2^Department of Epidemiology and Population Health, American University of Beirut, Lebanon; ^3^Department of Biochemistry and Molecular Genetics, American University of Beirut, Lebanon; ^4^Center for Drug Discovery and Department of Anatomy, Cell Biology and Physiological Sciences, American University of Beirut, Lebanon

**Keywords:** thymoquinone, colorectal cancer stem cells, 5-Fluorouracil resistance, colonospheres, apoptosis

## Abstract

The high recurrence rates of colorectal cancer have been associated with a small population of cancer stem cells (CSCs) that are resistant to the standard chemotherapeutic drug, 5-fluorouracil (5FU). Thymoquinone (TQ) has shown promising antitumor properties on numerous cancer systems both *in vitro* and *in vivo*; however, its effect on colorectal CSCs is poorly established. Here, we investigated TQ’s potential to target CSCs in a three-dimensional (3D) sphere-formation assay enriched for a population of colorectal cancer stem/progenitor cells. Our results showed a significant decrease in self-renewal potential of CSC populations enriched from 5FU-sensitive and resistant HCT116 cells at 10-fold lower concentrations when compared to 2D monolayers. TQ decreased the expression levels of colorectal stem cell markers CD44 and Epithelial Cell Adhesion Molecule EpCAM and proliferation marker Ki67 in colonospheres derived from both cell lines and reduced cellular migration and invasion. Further investigation revealed that TQ treatment led to increased TUNEL positivity and a dramatic increase in the amount of the DNA damage marker gamma H2AX particularly in 5FU-resistant colonospheres, suggesting that the diminished sphere forming ability in TQ-treated colonospheres is due to induction of DNA damage and apoptotic cell death. The intraperitoneal injection of TQ in mice inhibited tumor growth of spheres derived from 5FU-sensitive and 5FU-resistant HCT116 cells. Furthermore, TQ induced apoptosis and inhibited NF-κB and MEK signaling in mouse tumors. Altogether, our findings document TQ’s effect on colorectal cancer stem-like cells and provide insights into its underlying mechanism of action.

## INTRODUCTION

Colorectal cancer (CRC) is the third most common cancer in both men and women and the third leading cause of cancer-related deaths in the United States [1]. However, most of the patients are diagnosed in late stages, and approximately 50% of them encounter metastatic progression [2]. For metastatic CRC, treatments typically include chemotherapy with conventional agents such as 5-fluorouracil (5FU). Since its discovery 50 years ago, 5FU has been the backbone of treatments for CRC, but with a reduced success rate of less than 30% [3]. The ineffectiveness of 5FU has been mainly limited by drug resistance [4, 5]. Most of the colorectal cancer-associated mortality stems from the recurrence and metastatic spread of chemoresistant cells to other vital organs, mainly the liver and lungs [6]. A complete understanding of all the players remains to be uncovered; however, the presence of chemotherapy-resistant cancer stem cells (CSCs) is one of the significant causes of tumor recurrence [7]. CSCs can self-renew [8] and are known to be resistant to chemotherapies such as 5FU or oxaliplatin [9]. Therefore, there is a need to develop practical therapeutic approaches that target CSCs and prevent relapse [10, 11].

In the development and discovery of new potential anticancer agents, growing interest is heading towards ‘safe’ and widely available molecules, prominently from plant extracts. Thymoquinone (TQ: 2-isopropyl-5-methylbenzo-1,4-quinone) is the primary active molecule of black seed essential oil, which has shown promising effects against cancer both *in vitro* and *in vivo* [12]. The ability of TQ to target nine of the ten hallmarks of cancer as well as its efficacy, selectivity against colorectal cancer and lack of toxicity to normal tissues makes it potentially interesting for colorectal cancer therapy [13]. TQ’s ability to inhibit colorectal cancer growth and invasion and induce cell cycle arrest and apoptosis in colorectal cancer cell culture and animal models have been documented by us and others [13–17]. TQ has been shown to inactivate the JAK/STAT signaling pathway by inhibiting STAT3 phosphorylation, reducing c-Src and JAK2 activity and attenuating the expression of STAT3 target gene products [18]. TQ is known to modulate Wnt signaling through GSK-3β activation, β-catenin translocation, and reduction of nuclear c-myc [19]. TQ was also found to activate p53, induce PARP cleavage, and reactive oxygen species production (reviewed in [20]). Comprehensive studies about TQ’s potential effect on colorectal CSCs are lacking [21]. Despite the promising anticancer activity of TQ, the main limitation for its clinical translation lies in its hydrophobicity, poor bioavailability and capacity to bind to plasma proteins [22]. Very few studies investigated the pharmacokinetic and pharmacodynamic characteristics of TQ. One study showed that TQ is reduced into hydroquinone by catalyzing liver enzymes [23] and was detected in the plasma of rats for up to 12 hrs post oral administration [24]. In rabbits, the absolute bioavailability of TQ upon oral administration was 58% with a lag time of 23 minutes, and 99% of TQ was bound to plasma proteins [25]. Identifying TQ binding targets *in vivo* and determining their distribution profile can greatly help in better understanding TQ’s pharmacological properties.

In our study, we focused on investigating TQ’s efficacy on human colorectal cancer HCT116 cells, which are sensitive and resistant to 5FU. The main aim was to study the effect of TQ on targeting the self-renewal capacity of colorectal CSCs enriched from the parental and 5FU-resistant cell lines using the advanced three dimensional (3D) culture sphere-formation and propagation assay. *In vitro* and *in vivo* studies revealed the significant inhibitory potential of TQ on colorectal cancer cells with stem-like properties, which was found to be mainly mediated by induction of apoptosis. Our study documents TQ’s promising effect on CRC cancer stem-like cells both *in vitro* and *in vivo*.

## RESULTS

### TQ reduces the viability of 5FU-sensitive and resistant HCT116 human colorectal cancer cell lines

**Figure 1:**
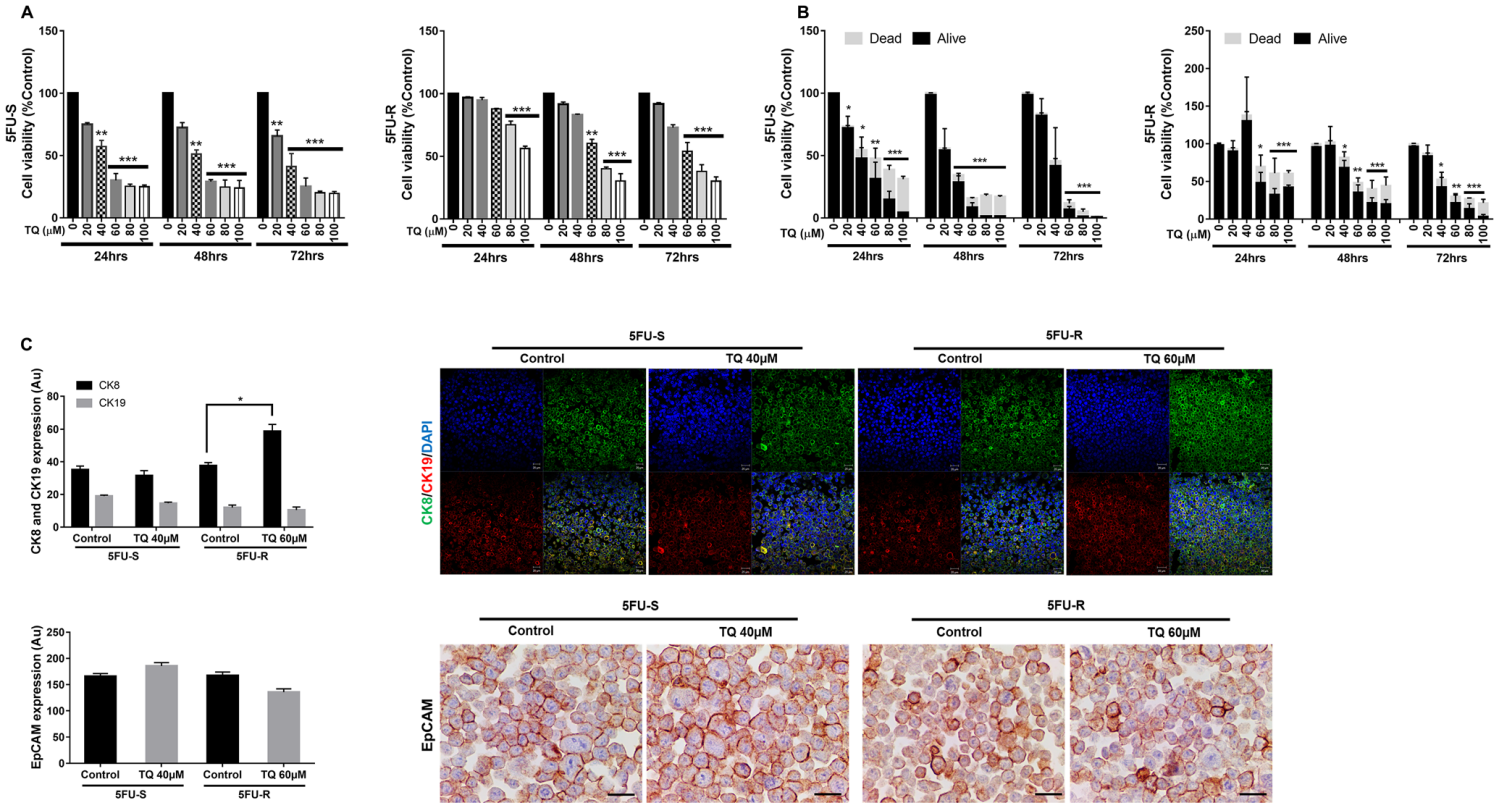
TQ reduces viability of 5FU-sensitive and 5FU-resistant HCT116 colorectal cancer cells. After incubation of 5FU-S and 5FU-R HCT116 colorectal cancer cells for 24, 48 and 72hrs with or without TQ, cell viability was determined using MTT assay (**A**) and Trypan blue dye exclusion assay (**B**). Results are expressed as percentage of the studied group compared to its control. Data represent an average of three independent experiments. The data are reported as mean ± SD for MTT and mean ± SEM for Trypan blue assay (^*^ *P* < 0.05; ^**^ *P* < 0.01; ^***^ *P* < 0.001). (**C**) 5FU-S and 5FU-R HCT116 colorectal cancer cells treated or not with 40 and 60 µM TQ respectively were immunofluorescently stained for CK8 and CK19 and immunohistochemically stained for EpCAM. Quantification and representative images are shown. Scale bar for immunofluorescent images is 20 µm and for immunohistochemistry is 100 µm.

Our first objective was to investigate the *in vitro* effect of TQ on the growth of HCT116 5FU-sensitive and resistant colorectal cancer cell lines cultured in 2D monolayers. MTT results showed a precise time- and dose-dependent reduction in viability in response to TQ. In the 5FU-sensitive cell line, the IC_50_ of TQ at 48 hrs and 72 hrs was ~40 µM (Figure 1A). In 5FU-resistant cells, the inhibitory effect of TQ commenced at a concentration of 60 µM at 48 hrs, decreasing cell viability by 40% (Figure 1A). The maximum percentage of reduction in viability at 72 hrs in the sensitive cell line was 80–85% compared to 70–75% in the resistant cell line. These results were consistent with Trypan blue exclusion assay (Figure 1B) and with the changes in cell morphology and confluency following drug treatment in both cell lines. TQ’s effect on normal cells has been previously reported where we showed that TQ was non-toxic to FHs74Int human normal intestinal cells for doses up to 60 µM [26].

### TQ targets an enriched population of 5FU-sensitive and resistant human colorectal cancer stem-like cells

**Figure 2:**
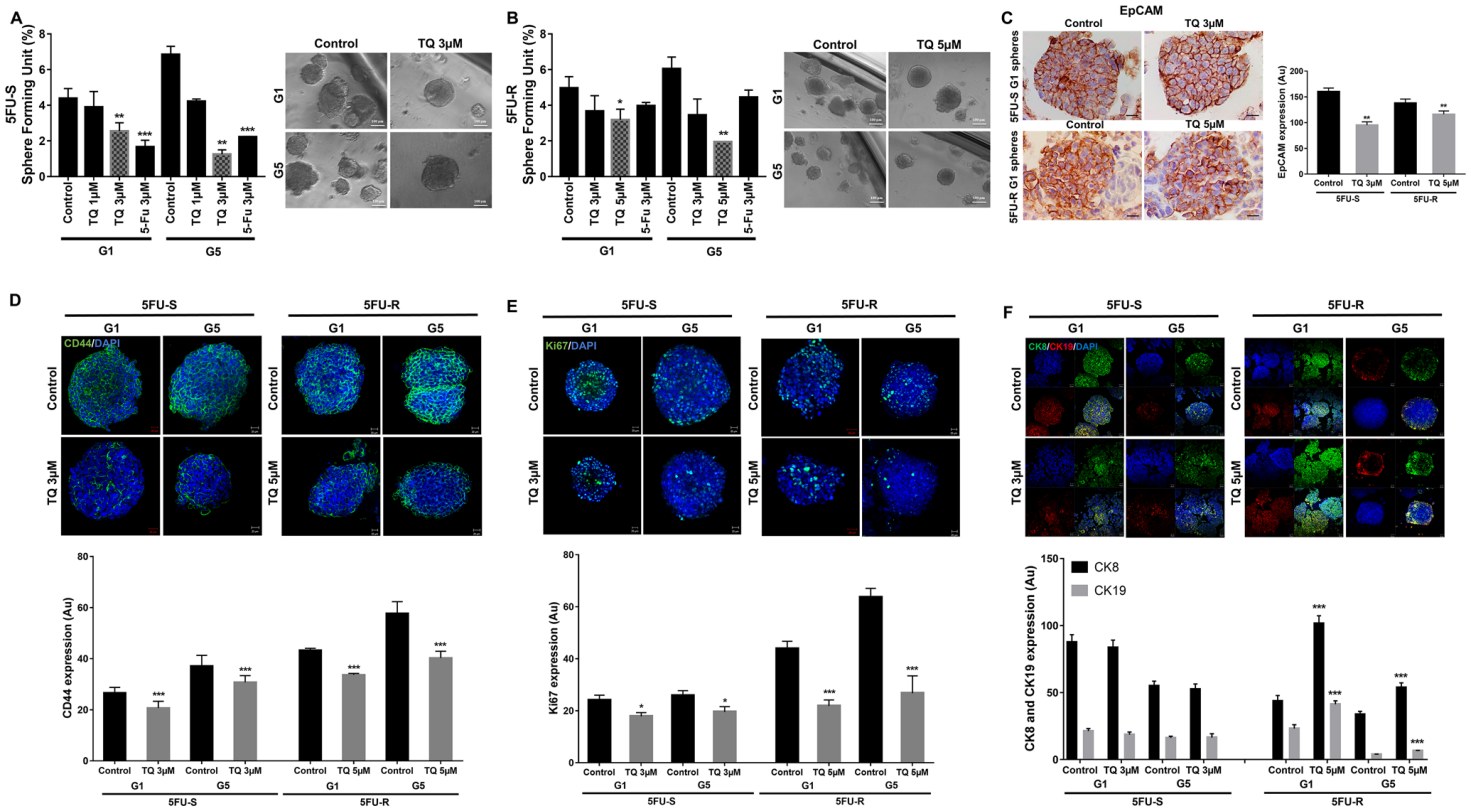
TQ reduces sphere-forming and self-renewal ability of colon cancer stem/progenitor cells. (**A**, **B**) Sphere forming unit (SFU) obtained from serially passaged colonopheres over five generations is shown under untreated conditions, TQ-treated (1, 3 and 5 µM) and 5FU-treated (3 µM) condition for 5FU-S (A) and 5FU-R (B) HCT116 derived spheres. SFU is calculated according to the following formula: SFU = (number of spheres counted ÷ number of input cells)*100. Colon CSCs were enriched from 5FU-S and 5FU-R HCT116 cell line and treated with either TQ (1, 3 and 5 µM) or media (control). Generated spheres are referred to as G1 (Generation 1) spheres. After each propagation, cells that were initially treated with TQ, 5FU or media (control) were seeded into separate wells. Spheres were propagated for five generations in duplicates for each condition. Data represent an average of three independent experiments and are reported as mean ± SEM (^*^ *P* < 0.05; ^**^ *P* < 0.01; ^***^ *P* < 0.001). Representative bright-field images showing the effect of TQ on SFU are shown next to the respective graphs. Images were visualized by Axiovert inverted microscope at 10× magnification and analyzed by Carl Zeiss Zen 2012 image software. Scale bar 100 µm. (**C–F**) Spheres were collected, fixed and stained for CD44, Ki67, CK8 and CK19 and EpCAM. Representative images were obtained using confocal and light microscopy and quantification of the intensity of EpCAM (C), CD44 (D), Ki67 (E) and CK8 and CK19 (F) stain in control and TQ treated 5FU-S and 5FU-R HCT116 G1 and G5 spheres was performed using Carl Zeiss Zen 2012 image software and ImageJ software for EpCAM intensity. Stain intensity was normalized to size. Data represent an average of three independent experiments and are reported as mean ± SEM (^*^ *P* < 0.05; ^**^ *P* < 0.01; ^***^ *P* < 0.001). Scale bar 20 µm.

Having established TQ’s inhibitory effect on both cell lines in 2D, we focused on investigating its potential inhibitory effect on targeting self-renewal capacity of colorectal CSCs enriched from 5FU-sensitive and resistant cell lines in 3D cultures using sphere formation and propagation assays. Cells that were able to form spheres in the first generation (G1) were collected and propagated by dissociating spheres into single cells and re-seeding the same number of cells (2000 cells/well). The assay was performed until the fifth generation (G5). In the 5FU-sensitive cells, treatment with 3 µM TQ significantly decreased the sphere formation ability up to G5 (Figure 2A). In the 5FU-resistant cells, on the other hand, most of the spheres treated with 3 µM 5FU remained viable up until the fifth generation, which confirms resistance to 5FU (Figure 2B). Interestingly, successive propagation and treatment of 5FU-resistant cells with 5 µM TQ significantly decreased sphere-forming unit (SFU) by a remarkable 70% after treatment (Figure 2B).

In addition to assessing the effect of TQ on self-renewal capacity, we investigated its effects on sphere size. Spheres were propagated for several generations with or without treatment, and at each generation, sphere sizes were determined (Figure 2A and 2B). TQ had no significant effect on the size of spheres derived from both 5FU-sensitive and resistant cells where the average diameter was around 100 µm with or without treatment.

To further study TQ’s effect on the enriched CSCs population, we analyzed the expression of the proliferation marker Ki67 and the stem cell markers CD44 and EpCAM. The immunofluorescent analysis showed that TQ treatment significantly decreased CD44 and Ki67 expression in 5FU-sensitive and 5FU-resistant HCT116 spheres by 1 to 2-fold (Figure 2D and 2E). Unlike 2D results (Figure 1C), immunohistochemical staining of spheres derived from both cell lines showed a significant reduction of EpCAM expression by ~1-fold (Figure 2C). This suggests that the reduction in sphere-forming ability is associated with decreased cellular proliferation and inhibition of key stem cell markers. Interestingly, TQ up-regulated cytokeratin epithelial markers, CK8 and CK19, in 5FU-resistant spheres and maintained an elevated expression of both in 5FU-sensitive spheres (Figure 2F), which could be indicative of reduced potential of epithelial-to-mesenchymal transition.

### TQ reduces invasion and migration ability of 5FU-sensitive and resistant-HCT116 colorectal cancer cells

**Figure 3:**
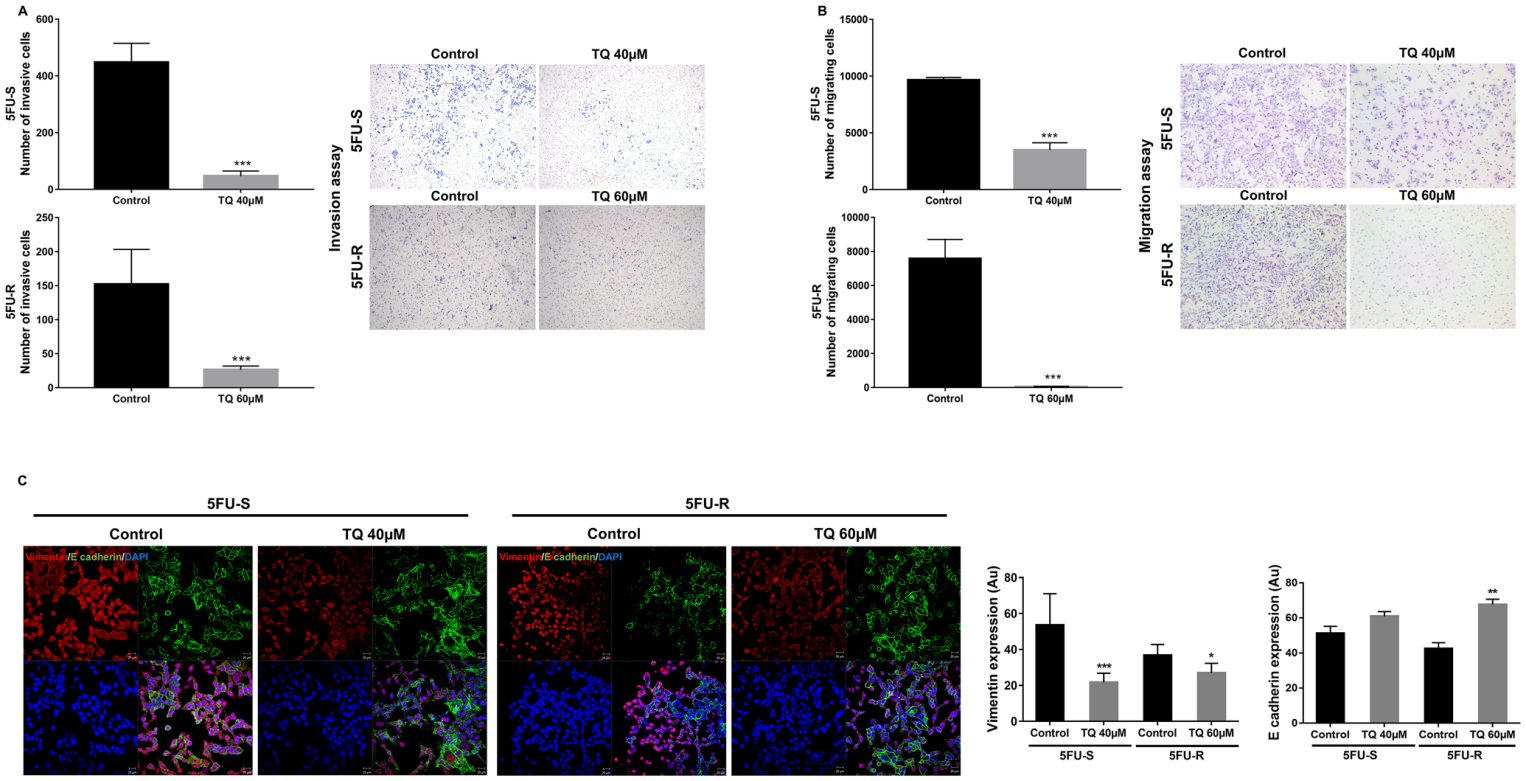
TQ reduces invasion and migration ability of 5FU-sensitive and 5FU-resistant HCT116 colorectal cancer cells. HCT116 cells were seeded onto the Matrigel-coated membrane (invasion assay) (**A**) or the uncoated membrane (migration assay) (**B**) in the top chamber of the transwell and were either treated or not with 40 and 60 µM TQ respectively in the presence of FBS in the lower chamber. Cells that migrated/invaded to the lower chamber after 48hr were fixed with methanol, stained with H&E, counted and represented as number of migrating/invading cells compared to the control. Data represent an average of three independent experiments. The data are reported as mean ± SEM (^*^ *P* < 0.05; ^**^ *P* < 0.01; ^***^ *P* < 0.001). (**C**) Representative confocal images and quantification of vimentin and E cadherin expression in 5FU-S and 5FU-R HCT116 colorectal cancer cells treated or not with 40 and 60 µM TQ, respectively. Data represent an average of three independent experiments and are reported as mean ± SEM (^*^ *P* < 0.05; ^**^ *P* < 0.01; ^***^ *P* < 0.001). Scale bar for immunofluorescent images 20 µm.

To study the possible mechanism of inhibition of stemness observed by the substantial reduction in colonosphere formation, we investigated the effect of TQ on cell migration and invasion, two phenotypes that are associated with the progression to metastasis. TQ treatment decreased cell invasion, whereby the invasion ability of cells in response to FBS was significantly reduced by more than 3-fold compared to the control (Figure 3A). Also, TQ significantly inhibited cell migration ability of 5FU-sensitive (~3-fold) and resistant HCT116 cells (~200-fold) compared to the control at 48 hrs (Figure 3B). This reduction in cell migration and invasion in response to TQ correlated with a significant downregulation in vimentin expression (1.5 to 2-fold decrease), an intermediate filament protein that is expressed in mesenchymal cells, and upregulation in E cadherin (~1.5-fold), an epithelial marker, in both 5FU-sensitive and resistant cells (Figure 3C). In addition, TQ up-regulated CK8 in 5FU-resistant cells when compared to control and maintained an elevated expression of both CK8 and CK19 in sensitive and resistant cells (Figure 1C). Collectively, these results suggest that TQ has a high inhibitory effect on colorectal cancer cell migration and invasion.

### TQ induces apoptosis and DNA damage in colorectal cancer stem/progenitor cells

**Figure 4:**
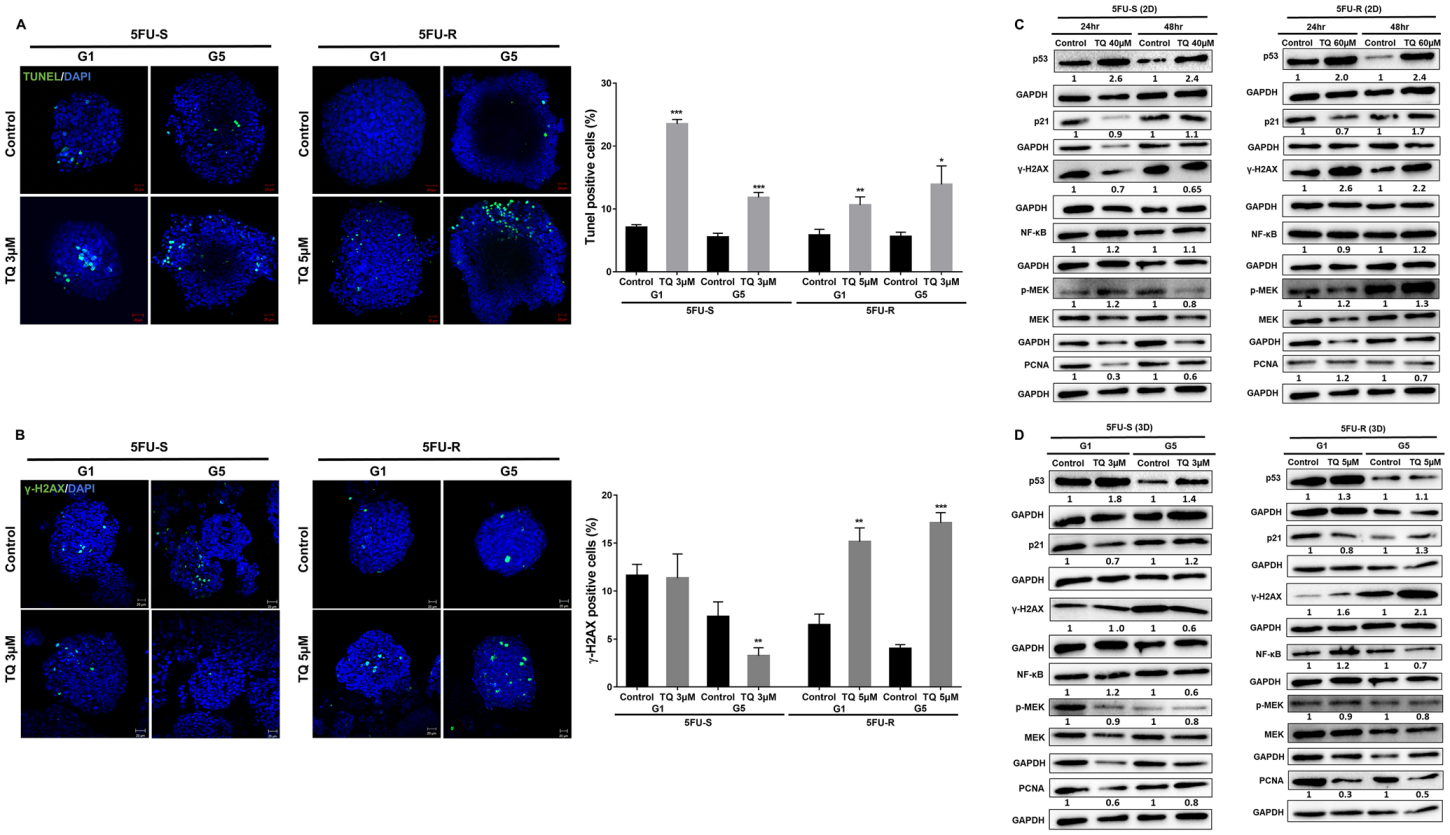
TQ induces apoptosis and DNA damage in colon cancer stem/progenitor cells. (**A**) Representative images of control and TQ treated 5FU-S and 5FU-R HCT116 G1 and G5 spheres after TUNEL staining. Scale bar 20 µm. TUNEL positive cells were counted and represented as mean percentage ± SD (^*^ *P* < 0.05; ^**^ *P* < 0.01; ^***^ *P* < 0.001 significantly different from control. (**B**) Representative images of γ-H2AX staining in control and TQ-treated 5FU-S and 5FU-R HCT116 G1 and G5 spheres. Scale bar 20 µm. γ-H2AX positive cells were counted and are represented as mean percentage ± SEM (^*^ *P* < 0.05; ^**^ *P* < 0.01; ^***^ *P* < 0.001 significantly different from control). (**C**, **D**) Analysis of p53, p21, γ-H2AX, NF-κB (p65), p-MEK and PCNA protein expression in 5FU-S and 5FU-R HCT116 cells and spheres during TQ treatment. Fold expression changes normalized to GAPDH and total MEK in case of p-MEK expression are given below the blots.

As mentioned previously, TQ caused a significant reduction in sphere number but not size, suggesting the involvement of a cell death mechanism. To determine TQ’s mechanism of action, we performed TUNEL staining on 5FU-sensitive and resistant HCT116 G1 and G5 spheres (Figure 4A). TQ treatment led to increased TUNEL positivity, indicating that the diminished sphere forming ability in TQ-treated colonospheres was in part due to the induction of apoptosis. In the TQ-treated spheres, mean apoptotic index estimated by TUNEL was 11.3% and 11.8% as compared to 8% and 5.5% in control G1 and G5 5FU-sensitive spheres, respectively. In 5FU-resistant spheres, mean apoptotic index estimated by TUNEL was 8% and 14% in TQ-treated spheres as compared to 0.5% and 5.6% in control G1 and G5 spheres, respectively. Analysis of p53 protein expression in 5FU-sensitive and 5FU-resistant 2D cells and 3D spheres during TQ treatment showed up-regulation further confirming apoptosis induction (Figure 4C, 4D). This was also associated with an upregulation in p21 expression (Figure 4C, 4D). Western blot analysis also showed a decrease in NF-κB, PCNA and p-MEK expression especially in 3D colonospheres (Figure 4D).

An early cellular response to double-strand breaks is the phosphorylation at Ser139 of a subclass of eukaryotic histones, H2AX. To study TQ’s effect on inducing DNA damage, we studied the expression of H2AX. Interestingly, TQ caused a dramatic increase in the amount of H2AX protein mainly in 5FU-resistant cells and spheres (Figure 4B, 4C) indicating a role of DNA damage pathway in these cells in response to TQ.

### TQ inhibits tumor growth in mice injected with 5FU-sensitive and resistant HCT116 spheres

To experimentally prove that the HCT116 population derived from spheres is enriched with cells having stem-like properties, we assessed their tumorigenic potential in mice. NOD-SCID mice were used for the 5FU-sensitive cell line as they easily showed tumor development. 5FU-resistant cells failed to develop tumors in NOD-SCID mice, so NOD/Shi-scid IL2rgamma^null^ (NOG) mice were used. The number of spheres needed for tumor development was optimized by serial dilution. 100 5FU-sensitive and 250 5FU-resistant spheres induced tumor development in 4 and 8 weeks in NOD-SCID and NOG mice, respectively (data not shown). We have previously reported that intraperitoneal injections of TQ at doses up to 20 mg/kg are not toxic to mice and significantly delay tumor growth in a xenograft model of 5FU-sensitive HCT116 colorectal cancer [27].

**Figure 5:**
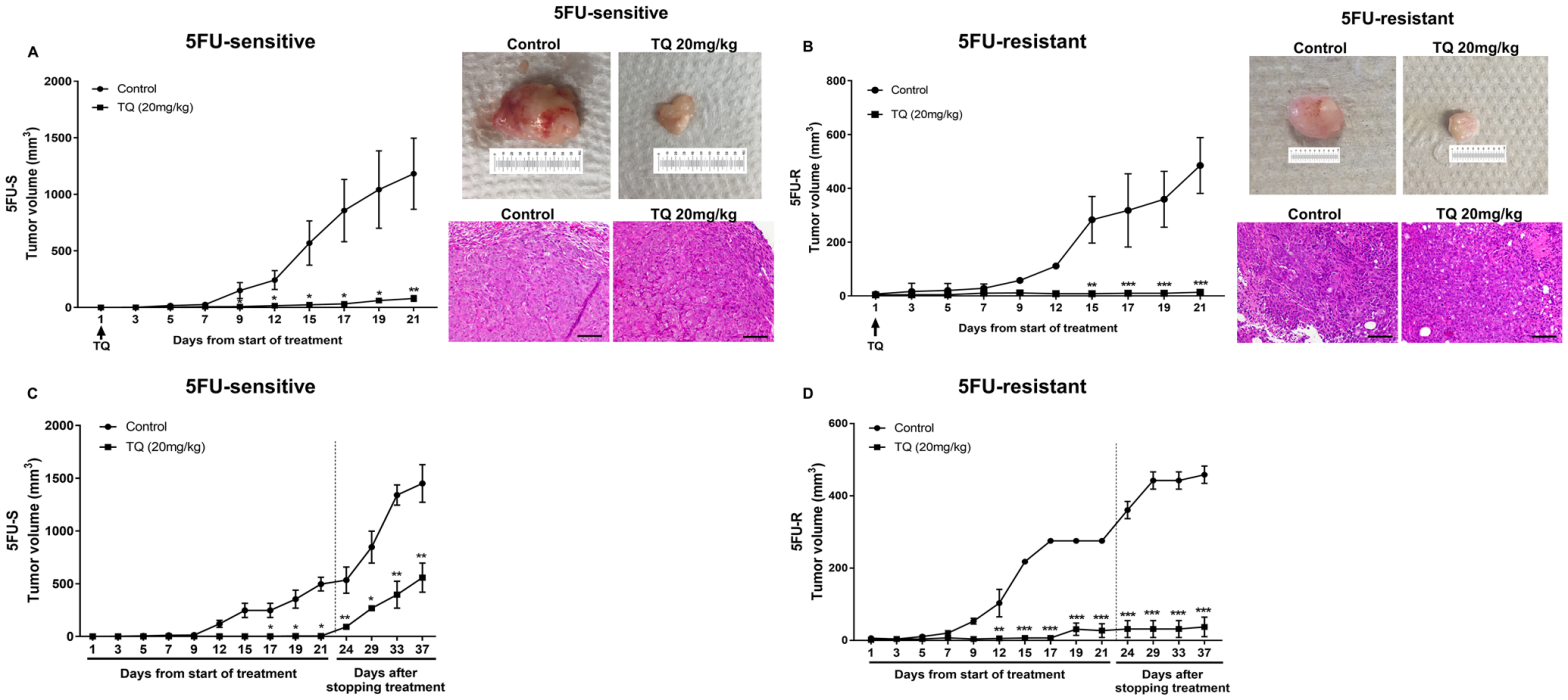
TQ reduces tumor growth in NOD-SCID and NOG mice. (**A**, **B**) NOD-SCID mice (8 mice/group) (A) were injected with 100 5FU-sensitive HCT116 G1 spheres and NOG mice (5 mice/group) (B) were injected with 250 5FU-resistant HCT116 G1 spheres and tumor progression was monitored. Tumor volume during 21 days of i. p. treatment (3×/week) with either 20 mg/kg TQ or 10% methanol in physiologic saline was reported. *P* < 0.05 between TQ and vehicle (control) treated animals. Representative images of control and TQ-treated mice at day 21 and H&E stain of tumor tissues are shown. (**C**, **D**) Average tumor volume of control and TQ treated mice (*n* = 3) during and after stopping treatment for 2 weeks was monitored in NOD-SCID and NOG mice and showed an increase in volume that was still significantly different from control untreated group.

To test the effect of TQ on targeting an enriched population of cells with stem-like properties *in vivo*, we injected two groups of NOD-SCID mice with 100 spheres derived from HCT116 sensitive cell line and two groups of NOG mice with 250 spheres derived from HCT116- resistant cell line. One group acted as a control, and the other group was treated with TQ at a dose of 20 mg/kg body weight [27] three times per week for 21 days by intraperitoneal injections when a palpable tumor was observed. TQ significantly inhibited tumor growth in these mice when compared to control group (Figure 5A, 5B). At the end of the treatment period, the average tumor volume was 1182 mm^3^ and 485 mm^3^ in the control group, while it was 79 mm^3^ (*P* < 0.01) and 14 mm^3^ (*P* < 0.001) in TQ treated mice injected with 5FU-sensitive and 5FU-resistant spheres, respectively (Figure 5A, 5B). A dose of 20 mg/kg TQ did not affect the body weight or resulted in animal death (data not shown), indicating that this dose is not toxic. Interestingly, two weeks after stopping TQ treatment, the average tumor volume in the TQ treated group (558 mm^3^ in 5FU-sensitive and 37.5 mm^3^ in 5FU-resistant) was still significantly lower than that of the control group (1451 mm^3^ in 5FU-sensitive and 459 mm^3^ in 5FU-resistant) (Figure 5C, 5D).

**Figure 6:**
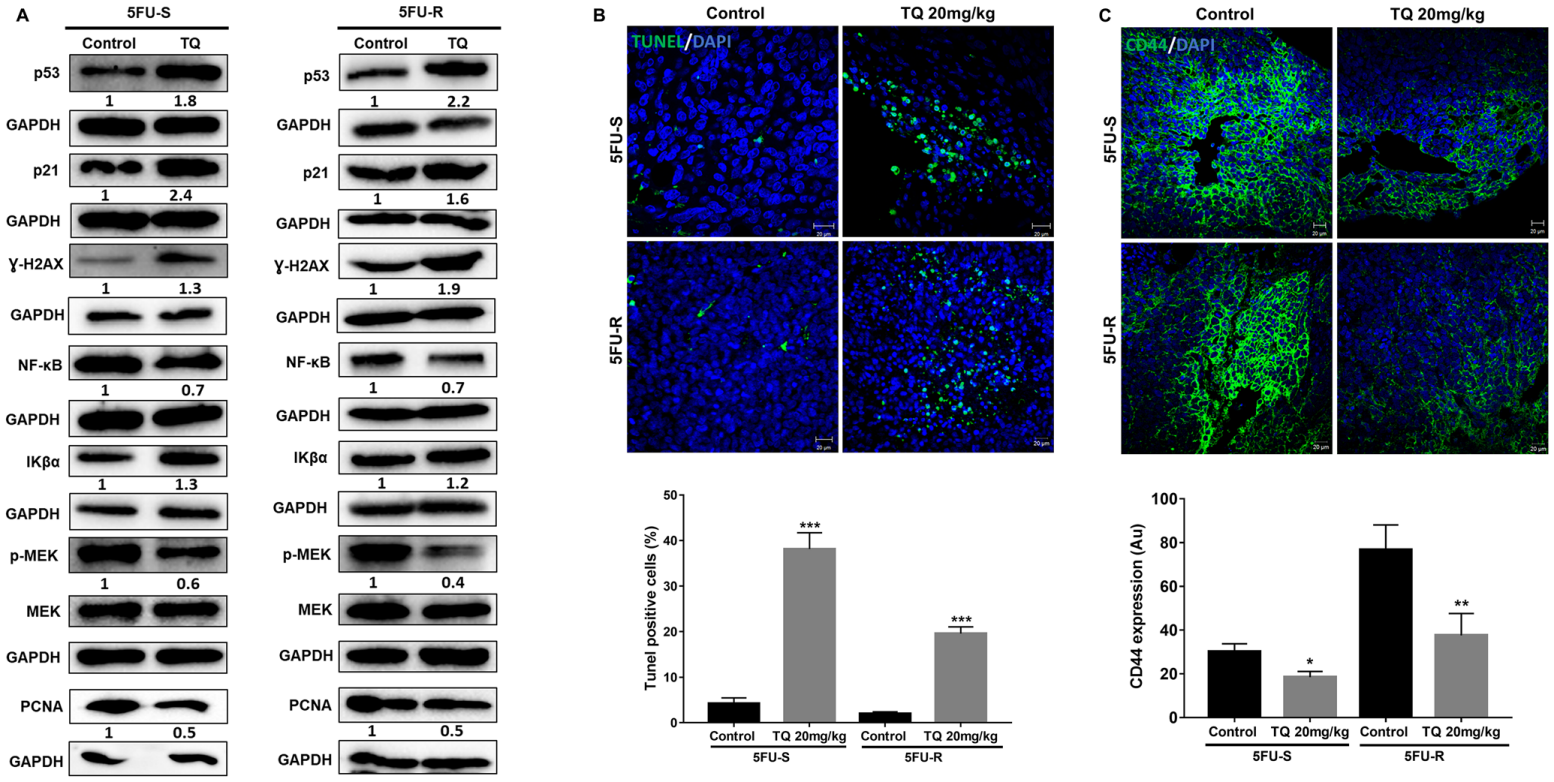
TQ induces apoptosis and reduces proliferation in NOD-SCID and NOG mice. (**A**) Analysis of p53, p21, γ-H2AX, NF-κB (p65), Iκβα, p-MEK and PCNA protein expression in control and TQ-treated tissues from NOD-SCID and NOG mice injected with 100 5FU-S and 250 5FU-R HCT116 G1 spheres. Fold expression changes normalized to GAPDH and total MEK in case of p-MEK expression are given below the blots. (**B**) Representative images of control and TQ treated tissues from NOD-SCID mice injected with 100 5FU-S and 250 5FU-R HCT116 G1 spheres after TUNEL staining. Scale bar 20 µm. TUNEL positive cells were counted and are represented as mean percentage ± SEM (^*^ *P* < 0.05; ^**^ *P* < 0.01; ^***^ *P* < 0.001 significantly different from control). (**C**) Representative confocal images of CD44 expression in control and treated tissues from NOD-SCID mice injected with 100 5FU-S and 250 5FU-R HCT116 G1 spheres. Images were analyzed and quantified by Carl Zeiss Zen 2012 image software.

The diminished tumor size in TQ-treated xenografts was in part due to the induction of cell death, as shown by increased TUNEL positivity (Figure 6B). In the TQ-treated group, average apoptotic index estimated by TUNEL was 51.6% and 20% as compared to 4.2% and 2% in vehicle controls in mice injected with 5FU-sensitive and 5FU-resistant spheres, respectively. Similar to *in vitro* results, the stem cell marker CD44 was also decreased in mouse tumor tissues upon TQ treatment (Figure 6C). Western blot analysis showed upregulation of p53, p21, γ-H2AX and the NF-κB inhibitor Iκβα, and downregulation of the proliferation markers PCNA, NF-κB (p65), and p-MEK in tumor tissues of TQ-treated mice (Figure 6A), similar to *in vitro* 3D results.

## DISCUSSION

The current study was designed to investigate the effect of TQ on targeting the self-renewal capacity of colorectal CSCs and its underlying mechanisms of action in 5FU-sensitive and resistant HCT116 cell lines *in vitro* and in xenograft mouse models. Sphere formation assay was used to enrich for colorectal CSCs as no consensus has been reached on the universal markers that define colorectal CSCs [28].

5FU remains to be the standard chemotherapy for metastatic CRC; however, cardiotoxicity and drug resistance limit its effectiveness. Various factors contribute to 5FU resistance which include a) genetic and epigenetic modifications within the cell itself, b) cell cycle and signaling pathway perturbations, c) or decreased drug delivery to cancer cells either by increased efflux out of the cell, decreased uptake or change in enzymes involved in metabolism [29]. Recent studies have also attributed colorectal cancer 5FU resistance to a population of cells with stem-like properties referred to as colorectal CSCs. Like other conventional cytotoxic chemotherapies, 5FU targets the rapidly dividing cells while sparing the quiescent or slowly cycling CSCs, thus enriching for the rare subsets of colorectal CSCs [30]. Therefore, identifying new therapeutic approaches that target CSCs is of high importance to prevent relapse. The natural compound TQ has shown promising antitumor activities against various cancer types [20]. In line with these studies, we have demonstrated that TQ exhibited anti-neoplastic effects by reducing the viability of 5FU-sensitive and resistant HCT116 cell lines in a time- and dose-dependent manner and decreased the expression of the proliferation marker Ki67.

Many studies have reported the promising potential of co-administering TQ with traditional chemotherapeutic agents. TQ/5FU combination has been shown to enhance 5FU action and to chemosensitize cancer cells to 5FU induced cell death in early stages of colorectal carcinogenesis in rats and in gastric cancer cells [31, 32]. However, studies tackling TQ’s effect on CSCs are limited. Ndreshkjana et al. (2018) have recently reported that the combination of 5FU and TQ and their hybridization through esterification (SARB hybrid) targets stem cell gene signature in colorectal cancer cells [21]. Here we showed that TQ exhibits a strong inhibitory effect on the self-renewal potential of CSC populations enriched from 5FU-sensitive and resistant HCT116 cells demonstrating that CSCs, which are resistant to chemotherapy compared to the bulk of the tumor cells, are selectively and effectively targeted by TQ. This reduction in sphere-forming ability correlated with the observed decrease in the expression of the stem cell markers CD44 and EpCAM. It is important to note that CD44 expression was more prominent in 5FU-resistant cell line when compared to 5FU-sensitive suggesting its role in stemness and resistance. CD44 is a transmembrane glycoprotein highly expressed in almost every cancer cell and is crucial in tumor initiation and colonosphere propagation *in vitro* [33]. CD44 is known to have a multifunctional role in many cellular processes, like survival, growth, and differentiation, and may regulate stemness in CSCs [34]. Our results showed that TQ treatment significantly reduced CD44 expression in CSCs population enriched from 5FU-sensitive and resistant cells both *in vitro* and *in vivo*.

TQ has been shown to induce apoptosis by modulating several types of players including generation of reactive oxygen species [26], up-regulation of apoptotic mediators, interference with angiogenesis, metastasis and DNA damage markers [20, 35]. To understand the observed reduction in sphere-formation, we checked for apoptosis induction and activation of DNA damage markers in spheres derived from the two cell lines. TQ treatment led to increased TUNEL positivity and upregulation of p53 and p21, indicating that the diminished sphere-forming ability in TQ-treated colonospheres was due to the induction of apoptotic cell death. H2AX is a member of the histone H2A family and is one of the first molecules to be phosphorylated at serine 139 (γ-H2AX) in response to double-strand DNA breaks. This phosphorylation mediates the recruitment of repair factors to the damaged DNA sites [36]. H2AX has been proposed as a factor to assess response to treatment, and several agents and chemotherapeutic drugs used in colorectal cancer treatment have been shown to increase γ-H2AX, including oxaliplatin, sorafenib, valproic acid, and oncolytic adenovirus [37, 38]. The response to DNA damage results in either cell cycle arrest, to allow the lesions to be repaired, or in p53-dependent and independent apoptosis [39, 40]. In this study, we showed that TQ downregulated γ-H2AX in 5FU-sensitive cells, which could suggest that TQ has a low genotoxic potential in these cells since it induced p53 activation with minimal DNA damage response [41]. In addition, γ-H2AX plays an essential role in the process of DNA repair through the recruitment of DNA repair proteins such as 53BP1, RAD51, BRCA1, and MDC1 to the damage sites [42]. Therefore, this decrease in γ-H2AX could indicate reduced DNA repair in malignant cells, which enhances their sensitivity to TQ. In contrast, γ-H2AX was remarkably upregulated by TQ in 5FU-resistant cells, indicating activation of DNA damage response, which may generate a positive feedback loop that enhances p53 activity. DNA damage can cause various post-translational modifications on p53 that can enhance its ability to activate target genes and promote apoptosis [41] thus increasing the susceptibility of the chemo-resistant malignant cells to apoptosis by TQ. γ-H2AX was upregulated in both 5FU-S and 5FU-R tumors, but this upregulation was more pronounced in 5FU-R tumors. Similar to *in vitro* results, p53 and p21 were also upregulated in these tumors suggesting apoptosis induction in response to TQ-induced DNA damage.

The ability of cancer cells to metastasize to vital organs is a major cause of cancer mortality [43]. TQ has been shown to inhibit migration and invasion of cancer cells by targeting epithelial to mesenchymal transition (EMT) markers Twist and E cadherin [44–46]. TQ was also shown to inhibit bone metastasis of breast cancer cells through abrogation of the CXCR4 signaling axis [12]. In accordance with these findings, our results also demonstrated that TQ significantly decreased the migration and invasion ability of 5FU-sensitive and resistant HCT116 cells, suggesting its role in inhibiting metastasis. CK8 and CK19 are members of the intermediate filament-forming proteins of epithelial cells. Several studies have provided evidence for active keratin involvement in cancer cell invasion and metastasis, as well as in treatment responsiveness [47]. Reduced expression of CK8 and CK20 has been associated with an increased transition in epithelial to mesenchymal cells in CRC [48, 49]. In this study, staining for CK8 and CK19 showed an increase in expression upon TQ treatment when compared to control mainly in the resistant cell line suggesting a protective role of TQ against metastasis.

To experimentally prove that the derived spheres are enriched with cells having stem-like properties, we injected a group of mice with HCT116 cells cultured in 2D monolayers and another group of mice with spheres. The validity of our model will be confirmed if the injected spheres show a higher tumor initiation capacity than 2D monolayer cells. Indeed, the injection of spheres derived from HCT116 sensitive cell line and not the 2D equivalent cell density into NOD-SCID immunocompromised mice resulted in tumor development, suggesting that spheres are rich in cells with stem-like properties. Treatment with 20 mg/kg body weight of TQ was able to inhibit tumor growth in NOD-SCID mice injected with 5FU-sensitive spheres and NOG mice injected with 5FU-resistant spheres and tumor volume in TQ-treated group remained significantly lower than that of control after stopping treatment for two weeks, indicating a relatively potent inhibitory effect of TQ on tumor growth. Interestingly, TQ’s effect on 5FU-resistant induced tumor volume was irreversible when compared to 5FU-sensitive tumor volume after stopping treatment for two weeks suggesting a promising effect of TQ on the enriched population of chemoresistant colorectal CSCs that is majorly responsible for tumor recurrence. This effect could be further enhanced with long-term exposure to TQ. Studies on the anticancer therapeutic potential of TQ and its safety profiles in humans are very limited and its pharmacologically relevant doses in animal or human blood have not been determined. In a Phase I clinical trial, TQ was found to be well tolerated at doses up to 10 mg/kg/day but no significant anticancer activity was observed at this dose [50]. A recent Phase II clinical study evaluating the effect of 100 mg and 200 mg TQ on oral potential malignant lesions is currently registered; however, it is still not open for participant recruitment (https://clinicaltrials.gov/ct2/show/NCT03208790?term=thymoquinone&rank=1). Nevertheless, several clinical trials testing the effect of *Nigella sativa* on various diseases including beta thalassemia major in children, dyslipidemia and arsenical keratosis have shown that it is not toxic in patients at doses up to 300 mg/day. (https://clinicaltrials.gov/ct2/results?cond=&term=nigella+sativa&type=&rslt=&age_v=&gndr=&intr=&titles=&outc=&spons=&lead=&id=&cntry=&state=&city=&dist=&locn=&strd_s=&strd_e=&prcd_s=&prcd_e=&sfpd_s=&sfpd_e=&lupd_s=&lupd_e=&sort=). The oral administration of TQ was found to be safe in several animal models [51]. The LD50 of TQ in mice and rats ranged between 57 and 104 mg/kg when injected intraperitoneally and reached 870 mg/kg when given orally (reviewed in Ref [52].), all of which are much higher doses than the effective anticancer dose of 20 mg/kg used in this study.

The macroscopically observed growth delay in HCT116 mouse xenografts was due to reduced proliferation of tumor cells and to drug-induced apoptosis as evidenced by decreased PCNA expression, upregulated p53 and p21 expression and enhanced TUNEL positivity in treated xenografts. To identify signaling pathways that might be involved in TQ’s effect on colorectal CSCs, we examined NF-κB (p65) and MEK/p-MEK expression. NF-κB is a crucial factor involved in the pathogenesis of inflammation mediated cancer through activation of genes and cytokines required for the induction of cellular proliferation [20] and has been shown to be regulated by TQ [53, 54]. In addition, MAPK signaling pathway is dysregulated in colorectal cancer, and various approaches for blocking signaling through this pathway have been studied [55]. We have previously shown that the inhibition of ERK pathway by MEK inhibitor PD98059 potentiated apoptosis induction by TQ [26]. We also documented that TQ directly binds to PAK1/ERK kinase complex, induces considerable conformational changes of PAK1 and interrupts its function as a scaffold for ERK1/2/MEK to recruit MEK to RAF at the membrane [56]. Importantly, MEK kinase was shown to induce NF-κB activation through the degradation of IκB-α, a major inhibitor of NF-κB [57]. Interestingly, TQ treatment reduced NF-κB (p65) and p-MEK and upregulated Iκβ-α expression in xenograft mouse tissues, suggesting a role for MEK as a signal mediator involved in Iκβ-α-induced NF-κB inhibition. Downregulation in NF-κB, p-MEK and PCNA was also observed *in vitro* especially in TQ-treated colonospheres highlighting the importance and advantage of 3D culture as a better mimic of *in vivo* environment.

Our study demonstrated that low concentrations of TQ could target CSCs enriched from 5FU-sensitive and resistant colorectal cancer HCT116 cell lines, suggesting a promising effect of TQ on chemoresistant cells. This effect when coupled with the apoptotic effects of TQ in human CRC cultures and xenografts indicates that this relatively non-toxic and inexpensive compound merits further clinical investigation.

## MATERIALS AND METHODS

### Cell culture conditions

Human colorectal cancer HCT116 5FU-sensitive (5FU-S) cells were purchased from ATCC and HCT116 5FU-resistant (5FU-R) cells were obtained from the group of Prof. Nadine Darwiche (American University of Beirut, Lebanon) [58]. Cells were cultured in their respective media either on Matrigel™ (BD Bioscience, Franklin Lakes, NJ, USA) or in 2D monolayer conditions. HCT116 cells were maintained in RPMI 1640 (Sigma-Aldrich, Germany) with 20 mM HEPES and L-Glutamine supplemented with antibiotics [1% Penicillin-Streptomycin (100 U/ml)] and 10% heat-inactivated fetal bovine serum (FBS) (Sigma-Aldrich, Germany). The cells were mycoplasma free and were maintained in an incubator at 37°C in a humidified atmosphere of 5% CO_2_ and 95% air.

### Drug preparation and treatment

Directly before use, fresh stocks of the purified synthetic compound TQ (Sigma-Aldrich: CAS: 490-91-5; 99.5% purity) in methanol and of 5FU (Sigma-Aldrich, Germany) in dimethylsulfoxide (DMSO) were prepared. Intermediate concentrations of the drugs were then made by serial dilutions from stock every two days during sphere formation assay. We assessed the sphere formation unit variation in response to different treatment conditions.

### MTT cell viability assay

5FU-sensitive and resistant HCT116 cells were plated in 100 µl complete medium in 96-well culture plates at a density of 10,000 and 12,000 cells/well, respectively. Cells were incubated overnight then treated in triplicates with various drug concentrations for 24, 48, and 72 hrs. Each experiment was repeated three times and in triplicate measurements. Cell viability was then assessed by MTT [3-(4, 5-dimethylthiazol-2-yl)-2, 5-diphenyltetrazolium bromide] that measures the ability of metabolically active cells to convert tetrazolium salt into violet formazan crystals. At specific time points, MTT reagent was added to each well and incubated at 37°C for 4 hrs. 100 µl isopropanol was used as a solubilizing solution to dissolve violet crystals. Consequently, MTT optical density (OD) was measured at a wavelength of 595 nm using ELISA reader (Multiskan Ex). Cell viability was expressed as a percentage of the control.

### Trypan blue viability assay

Supernatants containing dead cells were collected, and attached live cells were harvested by trypsin EDTA and added to the supernatant. The cell pellet was re-suspended in 100 µl media, and 50 µl of cell suspension was mixed with 50 µl of trypan blue and then live/dead cells were counted using a hemocytometer.

### Transwell migration assay

For the migration assay, 2.5 × 10^5^ 5FU-sensitive and 3.5 × 10^5^ 5FU-resistant HCT116 cells were seeded in a serum-free medium with or without treatment in the top chamber of 24-well inserts (pore size, 8 mm; Falcon), and a medium supplemented with serum was used as a chemo-attractant in the lower chamber. Cells were allowed to migrate through the membrane at 37°C in a 5% CO_2_ incubator for 24 and 48 hrs. Non-migratory cells in the upper chamber were then gently scraped off with a cotton-tip applicator. Migrating cells on the lower surface of the membrane were fixed and stained with Hematoxylin and Eosin (H&E). After staining, the total number of migrating cells was counted under the light microscope (10× objective) from six consecutive fields for each well.

### Transwell invasion assay

For the invasion assay, 2.5 × 10^5^ 5FU-sensitive and 3.5 × 10^5^ 5FU-resistant HCT116 cells were seeded in a serum-free medium with or without treatment in the top chamber onto the Matrigel™-coated membrane (24-well insert; pore size, 8 mm; Falcon), and a medium supplemented with serum was used as a chemo-attractant in the lower chamber. Each well was freshly coated with 100 µl of Matrigel™ (BD Bioscience, Franklin Lakes, NJ, USA) at a dilution of 1:10 in cold PBS and was then air-dried overnight before starting the invasion assay. Cells were allowed to migrate through the membrane coated with Matrigel™ at 37°C in a 5% CO_2_ incubator for 24 and 48 hrs. Non-migratory cells in the upper chamber were then gently scraped off with a cotton-tip applicator. Invading cells on the lower surface of the membrane were fixed and stained with H&E. After staining, the total number of invading cells was counted under the light microscope (10× objective) from six consecutive fields for each well.

### Sphere formation assay

HCT116 cells were able to generate spheres in non-adherent cultures. Single-cell suspension of HCT116 cell lines was counted, and a density of 2000 cells/well was suspended in cold Growth Factor Reduced Matrigel™/ serum-free RPMI-1640 medium (1:1) in a total volume of 50 µl [59]. Each experimental condition was performed in duplicate. The master mix of cells with Matrigel™ was circularly plated at the rim of the well of a 24-well plate and allowed to solidify in the incubator at 37°C for 45 minutes. Then 1 ml media with 5% FBS (with or without treatment) was added gently at the center of the well. Media or treatments were replenished every two days. Sphere counts and imaging were performed at day 9 and 13 of sphere culture, respectively, for the sensitive and resistant cell lines.

### Propagation assay

To enrich for the stem-like population of cells, the media was aspirated from the well and the Matrigel™ -containing spheres were digested by 500 µl dispase solution (Invitrogen, Carlsbad, CA, 1 mg dissolved in 1 ml RPMI-1640 incomplete medium) for 1 hr at 37°C. Spheres were collected and incubated in 0.5 ml Trypsin/EDTA at 37°C for 1–3 minutes. Single cells resulting from the dissociation of spheres were re-plated at the same density of 2000 cells/well in 24-well plates.

### 3D imaging of colonospheres

Spheres were grown then collected with cold RPMI media and centrifuged to washout all Matrigel debris. After centrifugation, spheres were fixed by formalin for 20 minutes. After washing with PBS three times, cells were permeabilized with 0.5% Triton X-100 for 30 minutes and blocked with sphere blocking buffer (0.1% BSA, 0.2% Triton X-100, 0.05% Tween-20, and 10% normal goat serum in PBS) for 2 hrs at room temperature. Spheres were washed and incubated overnight at 4°C with primary antibodies with blocking solution. Spheres were then washed with PBS and incubated with secondary antibody (Alexa fluoro 488 and Alexa fluoro 568) for 1 hr at room temperature. Finally, spheres were washed and mounted using the 5–7 µL anti-fade reagent Fluoro-gel II with DAPI (Abcam, Cambridge, UK). Fluorescent signals were captured using a Zeiss LSM 710 confocal microscope (Germany), and images were acquired and analyzed using the Zeiss LSM image software.

### TUNEL assay

Apoptosis was determined using the *In-Situ* Cell Death Fluorescein Detection Kit (11684795910, Sigma-Aldrich, Germany). For visualization of nuclei and mounting Fluoroshield Mounting Medium with DAPI (ab104139; Abcam, Cambridge, UK) was used, and samples were analyzed by a confocal microscope (LSM 710; Zeiss Germany).

### Western blot analysis

Spheres were grown with or without treatment then collected with cold RPMI media and centrifuged to wash out all Matrigel debris. Cells were plated in 100-mm tissue culture dishes and treated with 40 and 60 µM TQ for 48 hrs. Cellular protein extracts were prepared in RIPA lysis buffer (sc-24948, Santa Cruz, CA, USA). Protein extracts were quantified using the DC Bio-Rad Protein Assay (Bio-Rad Laboratories, Hercules, California, USA) according to the manufacturer’s protocol. Protein samples were mixed with 10% β-mercaptoethanol and 2× Laemmli Sample Buffer (Bio-Rad, CA, USA) for gel electrophoresis. An equal amount of protein lysate was separated on 12% SDS–PAGE for 2 hrs at 90 V then transferred onto 0.45 µm nitrocellulose membrane (Bio-Rad, CA, USA) in transfer buffer overnight at 40°C. Membranes were blocked with 5% skim milk in tris-buffered saline with 0.1% tween 20 (TBST) for 1 hr and then incubated overnight at 4°C with the primary antibody (all obtained from Santa Cruz, CA, USA; except GAPDH). Membranes were then washed three times with TBST and incubated with the diluted secondary antibody (Santa Cruz, CA, USA) for 1 hr at room temperature. Hybridization with GAPDH-HRP (6C5) (1:10,000–20,000, Abnova, #MAB5476) coupled antibody was performed for 30 minutes at room temperature as housekeeping gene. Target proteins were detected using the ECL system (Bio-Rad, CA, USA). Images were generated and quantified using ChemiDoc™ Imaging Systems (Bio-Rad, CA, USA).

### Histology and immunohistochemical analysis

Serial tissue sections (4 µm) were H&E stained and analyzed by an expert who was blinded for the treatment groups. Immunohistochemical staining was performed on paraffin-embedded spheres and mouse tumor tissues using antibodies against the Epithelial Cell Adhesion Molecule (EpCAM) and Ki67 (Santa Cruz, CA, USA). Slides were dried, dewaxed in xylene and rehydrated using a decreasing alcohol series. After blocking of endogenous peroxidase with H_2_O_2_, antigen retrieval was performed in 10 mM citrate buffer, pH 6. Subsequently, slides were blocked with Protein Block (Novolink Polymer Detection Kit, RE7150-K, Leica). Primary antibodies were incubated at 4°C overnight, followed by Post Primary and Novolink™ Polymer (Novolink Polymer Detection Kit, RE7150-K, Leica). Staining was visualized using 3,3-diaminobenzidine (DAB), and nuclear counterstaining was performed using hematoxylin (Novolink Polymer Detection Kit, RE7150-K, Leica Biosystems, Germany). Slides were dehydrated and embedded in Histofluid (6900002; Marienfeld, Lauda Koenigshofen, Germany). Images were recorded at 40× to 400× magnification using an Olympus BH-2 microscope and an Olympus E330 digital camera. The staining intensity was classified into 0 (no staining), 1+ (weak), 2+ (moderate), 3+ (strong), and the average of positively stained cells was recorded.

### Animal experiments

Six to eight week-old adult male NOD-SCID (injected with 5FU-sensitive cells) and NOG (injected with 5FU-resistant cells) mice were used. Mice were housed under optimum conditions of temperature set at 22 ± 2°C and light set at a 12 hrs light-dark cycle. Mice were kept in plastic cages covered with sawdust and had unrestricted access to a commercial mouse diet (24% protein, 4.5% fat, 4% fiber) and water. All animal studies were conducted using a protocol approved by the Institutional Animal Care and Use Committee of the American University of Beirut.

For tumor induction in mice, cells or spheres were suspended in 50 µl of the respective media, whereby cells or spheres were mixed with an equal volume of Matrigel. The mixture was subcutaneously injected into the flank of a group of mice. Animals were treated three times per week either with saline (control group) or TQ (20 mg/kg) by intraperitoneal injections when a palpable tumor was observed. Mice were daily monitored for signs of morbidity. Body weight recordings were carried out biweekly. Tumor volume was monitored every other day using Mitutoyo caliper.

### Statistical analysis

All statistical analyses (*t*-test and one-way ANOVA) were performed using GraphPad Prism 7 (version 7.0, GraphPad Software Inc., La Jolla, CA, USA). Normality of the data was confirmed using D’Agostino & Pearson and Shapiro-Wilk normality tests. In all statistical tests, the mean of treated groups was compared to the mean of control groups and a *p*-value < 0.05 was considered statistically significant.
